# Autistic children and adolescents with frequent restricted interest and repetitive behavior showed more difficulty in social cognition during mask-wearing during the COVID-19 pandemic: a multisite survey

**DOI:** 10.1186/s12888-022-04249-8

**Published:** 2022-09-14

**Authors:** Hiroki Tamon, Takashi Itahashi, Sosei Yamaguchi, Yoshiyuki Tachibana, Junya Fujino, Miki Igarashi, Makiko Kawashima, Riina Takahashi, Nozomi A. Shinohara, Yoshihiro Noda, Shinichiro Nakajima, Tomoya Hirota, Yuta Y. Aoki

**Affiliations:** 1 Division of Infant and Toddler Mental Health, Department of Psychosocial Medicine, National Center for Child Health and Development, Tokyo, Japan; 2 Graduate School of Medicine, Tohoku University, Miyagi, Japan; 3 Department of Functional Brain Imaging, IDAC, Tohoku University, Miyagi, Japan; 4 Medical Institute of Developmental Disabilities Research, Showa University, 6-11-11 Kita-karasuyama, Setagaya-ku, Tokyo, 157-8577 Japan; 5 National Center of Neurology and Psychiatry, Kodaira, Japan; 6 Department of Perinatal Mental Health, Shinshu University School of Medicine, Nagano, Japan; 7 Smart-Aging Research Center, IDAC, Tohoku University, Miyagi, Japan; 8 Department of Psychiatry and Behavioral Sciences, Graduate School of Medical and Dental Sciences, Tokyo Medical and Dental University, Tokyo, Japan; 9 Department of Psychology, Koishikawa Tokyo Hospital, Tokyo, Japan; 10 Department of Psychiatry, Aoki Clinic, Tokyo, Japan; 11 Department of Neuropsychiatry, Keio University School of Medicine, Tokyo, Japan; 12 Department of Psychiatry and Behavioral Sciences, University of California San Francisco, San Francisco, CA USA

**Keywords:** Asperger’s, Autism spectrum disorder, COVID-19 pandemic, CoRonavIruS Health Impact Survey (CRISIS), Mask-wearing, Restricted interest and repetitive behavior (RRB)

## Abstract

**Background:**

The public health measures enacted in order to control the coronavirus disease (COVID-19) pandemic have caused considerable changes to daily life. For autistic children and adolescents, adapting to the “new normal,” including mask-wearing, may be difficult because of their restricted interest and repetitive behavior (RRB) characteristics. We aimed to examine the relationships between RRB characteristics and the impact of mask-wearing on their social communications during the pandemic.

**Methods:**

We recruited participants with a clinical diagnosis of autism spectrum disorder based on DSM-5 diagnostic criteria from two outpatient clinics in Tokyo, Japan, between November 2020 and April 2021 using a convenience sampling methodology. As a result, the participants consisted of 102 children and adolescents (mean (*SD*) age = 11.6 (5.3)). We collected data on RRB characteristics frequency before and during the pandemic using the CoRonavIruS Health Impact Survey (CRISIS) – Adapted for Autism and Related Neurodevelopmental conditions (AFAR). We then conducted factor analyses to compute the RRB severity composite scores, which are divided into lower- (e.g., sensory seeking), and higher-order (e.g., restricted interest). We also investigated mask-wearing culture using a bespoke questionnaire, and using Spearman’s rank correlation analyses, we examined the relationships between before pandemic RRB characteristics, and the impact of mask-wearing on social communications during the pandemic.

**Results:**

We found that children and adolescents who exhibited lower-order RRB before the pandemic had difficulties in going-out with mask-wearing (rho = -0.25, *q* = .031), more challenges with mask-wearing (rho =  − 0.34, *q* = .0018), and difficulty in referring to others’ emotions while wearing masks (rho =  − 0.36, *q* = .0016). We also found an association between higher-order RRB before the pandemic and an uncomfortable sensation (rho =  − 0.42, *q* = .0002) and difficulties in referring to other’s emotions while wearing masks (rho =  − 0.25, *q* = .031).

**Conclusions:**

We revealed that various behaviors, such as sensory seeking, repetitive motor mannerisms and movements, and rituals and routines, undertaken before the pandemic could be important predictors of difficulties with mask-wearing and social communication for autistic children and adolescents during the pandemic. Caregivers and teachers wearing masks may need to provide extra support for social communication to autistic children and adolescents showing RRB characteristics frequently.

**Supplementary Information:**

The online version contains supplementary material available at 10.1186/s12888-022-04249-8.

## Background

Since its emergence, the COVID-19 pandemic has disrupted every aspect of daily life. To minimize the spread of the COVID-19 virus, governments worldwide declared states of emergency and mandated or recommended (depending on each country’s legal system) that people maintain social distance, wear masks, and stay at home [1, 2]. The rapid transformation of society into a new normal has caused a great deal of stress for many people, and has resulted in significant psychosocial impacts, including increased rates of depression and suicide in the general population [3, 4].

Autism spectrum disorder (ASD) is a developmental disability, of which the prevalence is approximately 2.3% of the general population [5]. Its core characteristics include difficulties in social communication, and restricted interest and repetitive behavior (RRB), including sensory characteristics and cognitive rigidity. Sensory characteristics can be both hyper- and hyposensitivity of the five senses, and are present in a certain proportion of autistic individuals [6]. Cognitive rigidity refers to the inability to adapt to changes or new environments. No established effective pharmacological intervention for these core ASD characteristics is currently available. Thus, one of the recommended approaches to mitigate RRBs in ASD is to provide a stable environment, such as establishing routines, which are suitable for autistic individuals [7]. However, under isolation mandates, maintaining routines has not always been possible [8]. Thus, the rapid transformation toward a new normal would have been particularly difficult for autistic individuals [9].

When they communicate, individuals on the autism spectrum spend more time looking at the mouth area of the face, and less time looking at the eye area of the face compared with neurotypical individuals [10]. Pazhoohi et al. demonstrated that neurotypical individuals with higher autistic characteristics were less accurate and less confident in identifying emotional face expressions during mask-wearing [11], but no study has focused on the impact of mask-wearing on individuals with ASD during the pandemic. [12]. While some studies did not support the increased mouth and diminished eye gaze hypothesis [13], accumulated research supports that individuals on the autism spectrum have difficulty reading eye gestures and expressions [13, 14]. Thus, the impact of mask-wearing on social cognition may be greater among individuals on the autism spectrum.

Sensory characteristics are one of four characteristics of RRB in the DSM-5: i.e. stereotyped or repetitive speech, excessive adherence to routines, fixated interests, and sensory characteristics. Because the sensory characteristics include tactile hypersensitivity, wearing masks would be particularly uncomfortable for autistic individuals. On the other hand, excessive adherence to routine includes rigid thinking, Thus, people with rigid thinking would have trouble with understanding others while wearing masks. We assume that the link between these two subcategories under RRB would affect social cognition through the influence of mask-wearing. On the other hand, we do not believe that links between the two other characteristics under the RRB umbrella and social cognition were influenced by mask-wearing because covering the mouth part of the face has no impact on these two characteristics.

In this context, we have examined the relationships between RRB characteristics and mask-wearing as well as mask-wearing and social cognition using the CoRonavIruS Health Impact Survey (CRISIS) – Adapted for Autism and Related Neurodevelopmental conditions (AFAR)**,** an internationally developed questionnaire designed for ASD. Because we assume that autistic children and adolescents would be more vulnerable to the transformation of society, we hypothesized that those with frequent RRB characteristics would show more difficulty with mask-wearing and social cognition. Additionally, although autistic individuals in all age group spend less time looking at the region of the eyes (reviewed in [15]), we focused on children and adolescents because they have more difficulty maintaining routines than do adults under pandemic public health measures [16, 17].

### Aim

We aimed to examine the relationships between RRB characteristics and the impact of mask-wearing on social communication among autistic children and adolescents during the pandemic.

## Methods

### Design and setting of the study

**Fig. 1:**
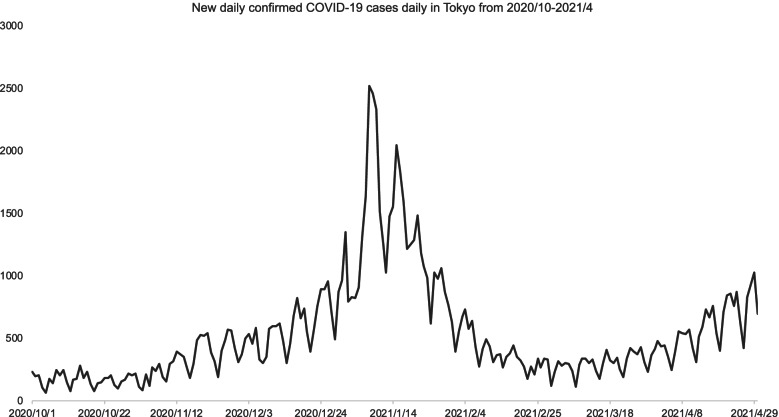
Trend of the number of daily confirmed COVID-19 cases in Tokyo during the survey period. Shows the line graph of the number of daily confirmed COVID-19 cases in Tokyo during the survey period

We recruited participants from the outpatient clinics of Showa University and the National Center for Child Health and Development, both located in Tokyo, Japan. These institutes are located in the western part of Tokyo. Since Tokyo is a large city, the socioeconomic status of residents varies greatly from region to region. Although socioeconomic status of individual participants was not fully obtained, we expect the participants to be of similar socioeconomic status because their addresses are geographically nearby one another. We contacted all clients with a clinical diagnosis of ASD based on DSM-5 diagnostic criteria who visited one of these institutes from November 2020 to April 2021. Figure 1 shows the line graph of the number of daily confirmed COVID-19 cases in Tokyo during the survey period. During the data collection period, the pandemic was overwhelming. Although the number of cases fluctuated during the research period, the public health measure had not changed during the research period. Because the metropolitan government did not require school shut down, many schools provided online classes.

### Characteristics of participants

Between November 2020 and April 2021, we contacted 127 children and adolescents. Among them, 104 answered the questionnaire, and two of these were excluded because their ASD diagnoses were not confirmed by an expert review. As a result, 102 autistic children and adolescents joined the study. Participants received a maximum of ¥1,000 gratuity for completing questionnaires. Frequencies and percentages of key demographic variables and COVID-related experiences are listed in Table 1. Additionally, we collected participants’ and caregivers’ educational levels as an alternative for socioeconomic status (SES). For children and adolescents, the educational levels were assessed using a 7-point Likert scale: 1: Not in school, 2: Preschool/Kindergarten, 3: Elementary School, 4: Junior High or Middle School, 5: High School, 6: College (University)/Vocational, and 7: Graduate School. For caregivers: the educational levels were also assessed using a 7-point Likert scale: 1: Some grade school, 2: Some high school, 3: High school diploma, 4: Some college or 2-year degree, 5: 4-year college degree or university graduate, 6: Some school beyond college, and 7: Graduate or professional degree. We took the maximum educational levels among parents as a representative SES for the family [18]. Most of the caregivers were mothers (*n* = 92, 90.2%) or fathers (*n* = 7, 6.9%) of autistic children and adolescents, while three did not indicate their relationship with the participants. The psychiatric and neurological comorbidities observed included attention-deficit/hyperactivity disorder (*n* = 28, 27.5%), learning disorder (*n* = 5, 4.9%), epilepsy (*n* = 8, 7.8%), and intellectual disabilities (*n* = 22, 21.6%). Relative to the low incidence rates of COVID-19 in Japan, it is not surprising that only two participants had a family member diagnosed with COVID-19, whereas 99 participants did not have any family members diagnosed with COVID-19 (one participant did not answer). One person had contact with a person with symptoms potentially related to COVID-19, whereas the rest had no contact with anyone who had symptoms or were diagnosed with COVID-19.

**Table 1:** Frequencies and percentages of key demographic variables and COVID-related experiences

Age	mean (*SD*)
Participants	11.6 (5.3)
Caregivers	45.7 (6.2)
**Sex**	***n* (%)**
Male	75 (73.5)
Female	27 (26.5)
**Relationship to the child**	
Mother	92 (90.2)
Father	7 (6.9)
NA	3 (2.9)
**Educational levels**	
**Participants**	
Not in school	10 (9.8)
Preschool/Kindergarten	15 (14.7)
Elementary School	37 (36.3)
Junior High or Middle School	14 (13.7)
High School	18 (17.7)
College (University)/Vocational	7 (6.9)
Graduate School	0 (0.0)
NA	1 (1.0)
**Caregivers**	
Some grade school	0 (0.0)
Some high school	0 (0.0)
High school diploma	28 (27.5)
Some college or 2-year degree	18 (17.7)
4-year college degree or university graduate	49 (48.0)
Some school beyond college	0 (0.0)
Graduate or professional degree	6 (5.9)
NA	1 (1.0)
**Psychiatric and neurological comorbidities**	
Attention-deficit/hyperactivity disorder	28 (27.5)
Learning disorder	5 (4.9)
Epilepsy or seizures	8 (7.8)
Obsessive compulsive disorder	1 (1.0)
Emotional or mental health problems such as depression or anxiety	16 (15.7)
Problems with alcohol or drugs	1 (1.0)
Intellectual disability	22 (21.6)
Other problems requiring special education services	4 (3.9)
Other neurodevelopmental conditions	2 (2.0)
Developmental delay	28 (27.5)
**Family member diagnosed with COVID-19**	
No	99 (97.1)
Yes	2 (2.0)
NA	1 (1.0)
**2-week exposure**	
None	101 (99.0)
Exposure to person with symptoms	1 (1.0)

*Abbreviations*: *ASD* Autism spectrum disorder, *COVID* Coronavirus disease of 2019, *NA* Not Answer

### Questionnaires

The CoRonavIruS Health Impact Survey (CRISIS) – Adapted for Autism and Related Neurodevelopmental conditions (AFAR) was used for this study [19]. This questionnaire, which is freely available online (http://www.crisissurvey.org/crisis-afar/), assesses daily life behaviors, other behaviors clinically relevant to autism including RRB, service changes which occurred during the pandemic, and COVID-19 worries. The survey has three versions depending on the participant’s age and reporter, such as a caregiver-report form for children and adolescents (3–21 years old), a self-report form for youth and adults (≥ 14 years old), and a caregiver-report form for adults. In the present study, we used the caregiver-report form for children and adolescents. This form consists of 93 questions. Some were Likert-type, whereas others were discrete variables or descriptive (please see details on the original version in English). Some of this study’s authors translated the English version into Japanese. The Japanese version was then back-translated by different authors to verify the quality of the translation. All language versions of the questionnaire are freely available online (http://www.crisissurvey.org/crisis-afar/. For the purpose of this study, we selected specific domains, including RRB characteristics, as detailed below.

Besides the CRISIS-AFAR, we added an original questionnaire which focused on the mask-wearing culture in Japan (CRISIS-AFAR-J). It included six CRISIS-AFAR-J questions as follows:

- Did your child find it difficult to wear masks and go out?
- Did your child feel an uncomfortable sensation while wearing a mask?
- Do you think your child finds it difficult to communicate because others are wearing masks?
- Do you think your child finds it difficult to recognize other people’s emotions because they are wearing masks?
- Do you think that your child’s wearing a mask makes it harder for people to hear him or her?
- Do you think your child finds it difficult to convey his or her emotions to others while wearing masks?

The answers for these questions were formulated in a Likert-type format: (1) yes, (2) somewhat yes, (3) I cannot say either, (4) somewhat no, and (5) no.

To further characterize the participants, we collected the Autism Spectrum Quotient and Social Responsiveness Scale scores for each participant (Table 2).

**Table 2:** Psychological properties of participants

	Mean (SD)
SRS (*n* = 26)	
Total	74.4 (16.3)
social awareness	64.9 (16.3)
social cognition	68.9 (15.8)
social communication	72.9 (14.5)
social motivation	61.7 (11.3)
autistic mannerisms	81.7 (22.2)
AQ (*n* = 41)	
total	26.7 (6.8)

*Abbreviations*: *AQ* Autism spectrum quotient, *SRS* Social responsiveness scales

### RRB characteristics frequency change

We focused on questions that measured RRB characteristics before and during the pandemic in the CRISIS-AFAR. These questions are listed in the Supplement. In the paired questions, the first asked about the participant’s behavior during the three months prior to the pandemic, and the second asked about the participant’s behavior during the previous two weeks (i.e., during the pandemic). To test the difference in questionnaire answers for RRB characteristics between before and during the pandemic, we utilized Wilcoxon matched-pairs signed rank tests.

### Statistical analyses

We computed the composite scores using answers to RRB questions in our data. The combinations of the answers were derived from the original CRISIS-AFAR Parent/Caregiver survey [19]. Briefly, in the original CRISIS-AFAR Parent/Caregiver survey, exploratory and confirmatory factor analyses (i.e., EFA and CFA) were performed on separate split-half datasets which were matched for demographic information, including sample, sex, child age, full-scale intelligence quotient, and primary DSM-5 diagnosis. In the EFA, the questions with resulting factor loading ≥ 0.3 were retained. Then, the CFA was subsequently performed only on the retained questions. Using the questions identified by these procedures, we calculated the composite scores in RRB. The RRB characteristics were divided into the lower-order RRB which includes sensory seeking, repetitive motor mannerisms/movements, and rituals and routines; and higher-order RRB characteristics which include requests to family members to maintain specific routines, rituals, and habits; as well as engaging in an activity related to a highly restricted and strong interest. To confirm the internal consistency of lower- and higher-order RRB metrics, we calculated Cronbach’s α coefficients. The lower- and higher-order RRB metrics exhibited α = 0.839 and α = 0.591, respectively. To further test the validity of CRISIS-AFAR, we conducted correlation analyses between the RRB standardized *t* scores of SRS and three items related to RRB characteristics in the CRISIS-AFAR.

### Association between hypersensitivity and mask-wearing

To examine the relationships between RRB and mask-wearing, we focused on the results of factor analyses: the lower- and higher-order RRB characteristics (see Results section). Spearmans’ rank correlation analyses were conducted between both the lower- and higher-order RRB characteristics and the mask-wearing impact data from the CRISIS-AFAR-J. Statistical significance was set at *q* < 0.05 after applying false discovery rate (FDR) correction for multiple comparisons.

### Follow-up analyses

To determine whether the association between lower-order RRB characteristics and mask-wearing impact was preserved in specific age groups, we divided the participants into two groups based on the age of 13. We chose this age because children older than 13 are typically junior high school students, while younger children attend elementary schools. Children at the age of 12 or younger were categorized as children, while those older were categorized as adolescents. We repeated the correlational analyses only on items showing statistically significant associations. Since we have a priori hypothesis that, for each group, there is an association in the same direction, *p*-values were computed in one-tailed and then FDR correction method was applied. Statistical significance was set to *q* < 0.05.

## Results

### Association between hypersensitivity and mask-wearing

Our analysis revealed that children and adolescents who exhibited lower-order RRB more frequently before the pandemic as derived from factor analysis of original CRISIS-AFAR data had more difficulties in going-out with mask-wearing (CRISIS-AFAR-J Question No. 1; rho = -0.251, *q* = 0.031) and more challenges with mask-wearing because of an uncomfortable sensation (CRISIS-AFAR-J Question No. 2; rho =  − 0.343, *q* = 0.0018). Additionally, autistic children and adolescents with more frequent observation of lower-order RRB characteristics before the pandemic showed more difficulty in recognizing others’ emotions while wearing masks (CRISIS-AFAR-J Question No. 4; rho =  − 0.356, *q* = 0.0016) (It should be noted that a negative correlation coefficient denotes a positive association because of the questionnaire design). We also found that a higher-order RRB was associated with an uncomfortable sensation during mask-wearing (CRISIS-AFAR-J Question No. 2; rho =  − 0.415, *q* = 0.0002) and difficulties in recognizing others’ emotions while wearing masks (CRISIS-AFAR-J Question No. 4; rho =  − 0.253, *q* = 0.031).

### Follow-up analyses

Both children and adolescent subgroups presented the same pattern as seen in the primary analysis. More specifically, children aged ≤ 12 also showed that the lower-order RRB more frequently presented more challenges in going-out with wearing mask (CRISIS-AFAR-J Question No. 1; rho = -0.280, *q* = 0.042), more challenges with mask-wearing because it was associated with an uncomfortable sensation (CRISIS-AFAR-J Question No. 2) (rho =  − 0.283, *q* = 0.042), and difficulty in referring to others’ emotions while wearing masks (CRISIS-AFAR-J Question No. 4) (rho =  − 0.365, *q* = 0.015). We did not observe statistically significant associations between higher-order RRB frequency and CRISIS-AFAR-J Questions (all *q* > 0.06). Adolescents aged ≥ 13 also demonstrated the association between lower-order RRB frequency and an uncomfortable sensation (CRISIS-AFAR-J Question No. 2; rho =  − 0.281, *q* = 0.042) while exhibiting the associations between higher-order RRB frequency and an uncomfortable sensation (CRISIS-AFAR-J Question No. 2; rho =  − 0.462, *q* = 0.004), and difficulty in referring to others’ emotions while wearing masks (CRISIS-AFAR-J Question No. 4; rho =  − 0.370, *q* = 0.015).

### Association between SRS and CRISIS-AFAR

The analyses showed that RRB standardized *t* scores for SRS were correlated with (1) repetitive motor mannerisms/movements (*r* = 0.611, *q* = 0.0007), (2) sensory-seeking behaviors (*r* = 0.705, *q* = 0.0001), and (3) rituals or routines (*r* = 0.659, *q* = 0.0003) as measured by the CRISIS-AFAR.

## Discussion

The present study examined the association between pre-pandemic RRB characteristics and the ramifications of mask-wearing by autistic individuals. We analyzed the data on children and adolescents obtained from the caregiver-report forms, and found that higher frequency of both lower- and higher-order RRB before the pandemic were associated with more difficulty wearing masks. Of note, autistic children and adolescents that presented more frequent lower-order RRB showed the greater levels of social communication difficulties during mask-wearing.

The present findings are in part consistent with those of a previous study [11]. Indeed, given that autistic people spend more time looking at the mouth than the eyes, it is reasonable to suggest that mask-wearing would make it more difficult to recognize others’ emotions [12]. In the present study, the questionnaire does not cover baseline social communication skills. Thus, we cannot specify that the association with the ramifications of mask-wearing is specific to RRBs, or can be applied to baseline communication skills. On the other hand, using the CRISIS-AFAR-J, we specifically asked about changes in social communication skills induced by mask-wearing. Thus, it is not likely that the present findings reflected the association between RRB and social communication impairment characteristics, which is confirmed by previous studies [20]. Namely, the current findings can be interpreted such that autistic children and adolescents with more RRB characteristics would be more vulnerable to the lifestyle changes induced by public health measures, and have more difficulty with social cognition. We are not aware of any study that specifically examined the association between RRB characteristics and adaptation to the new normal in autistic individuals, but given that one of the four sub-domains of RRB is insistence on sameness, people with more RRB characteristics are likely to have more difficulty dealing with changes to their lifestyle. Because pandemic related public health measures forced people to change their lifestyle, it is reasonably assumed that people with RRB characteristics that are affected by the public health measures are vulnerable to change induced by the public health measures. On the other hand, in terms of autistic people who were already isolated from society, public health measures did not have any influence on lifestyle regardless of the severity of their RRB characteristics. Future studies are needed to comprehensively reveal the associations between RRB characteristics, social cognition, and vulnerability to change.

### Limitations

The present findings need to be treated with caution. First, although the CRISIS-AFAR is a well-designed questionnaire which covers RRB and daily life behaviors, it does not measure ASD severity or establish diagnoses. Moreover, it does not address social communication difficulties, which means that we cannot verify that the present findings are directly specific to RRB or ASD severity. Second, the current questionnaire asked about characteristics three months prior to the pandemic, which means that the results may suffer from recall bias. Additionally, the present study adopted a caregiver-form for both CRISIS-AFAR (questionnaires about social cognition) and CRISIS-AFAR-J (questionnaires about mask-wearing). Although the consistency of the raters was one of the strengths of the study design, it should be noted that the pandemic caused rapid life changes and disruptions to routines which affected both the subjects and caregivers as well [21]. Thus, psychological stress, anxiety, and depression among caregivers may introduce bias into the current findings [22]. Third, because few COVID-19 studies have recruited children [23], one of the strengths of the current study is its focus on children. However, given that prior studies demonstrated the psychological impact of COVID-19 pandemic among adults [23], future studies are expected to examine whether the association between mask-wearing and social communication impairment is observed among adults. Furthermore, although the questionnaires were designed by experts, they were not validated in English or Japanese. Finally, because the questionnaires asked the caregivers to select the answers from the provided options, the sensitivity of the questionnaires to detect change was limited.

## Conclusions

We revealed that various behaviors such as sensory seeking, repetitive motor mannerisms and movements, and rituals and routines undertaken before the pandemic could be important predictors of difficulties with mask-wearing and social communication for autistic children and adolescents during the pandemic. Caregivers and teachers wearing masks may need to provide extra support for social communication to autistic children and adolescents showing RRB characteristics frequently.

## Supplementary Information

**Additional file 1.**

## Data Availability

The datasets generated and/or analyzed during the current study are not publicly available because they will be further analyzed, but are available from the corresponding author on reasonable request.

## Abbreviations

ASD: Autism spectrum disorder
RRB: Restricted interest and repetitive behavior
