# Supplementary material for: Autistic children and adolescents with frequent restricted interest and repetitive behavior showed more difficulty in social cognition during mask-wearing during the COVID-19 pandemic: a multisite survey
Source: BMC Psychiatry. 2022 Sep 14;22:608. doi: 10.1186/s12888-022-04249-8 (PMC9471034; doi:10.1186/s12888-022-04249-8)
Supplement: Supplementary file 1 — Additional file 1. [file 12888_2022_4249_MOESM1_ESM.docx]

Supplementary Information for “Autistic children and adolescents with frequent restricted interest and repetitive behavior showed more difficulty in social cognition during mask-wearing during the COVID-19 pandemic: a multisite survey” by Tamon et al.

**Supplementary Methods**

**CRISIS-AFAR questionnaires**

**RRB symptoms**

No. 51 (three months prior to the pandemic) and No. 75 (past two weeks of the survey):

**“How frequently did your child engage in repetitive motor mannerisms/movements (e.g., repetitive movements of the whole body, or just with their hands and fingers)”**

No. 52 (three months prior to the pandemic) and No. 76 (past two weeks of the survey):

**“How frequently did your child engage in sensory-seeking behaviors (e.g., visually inspecting things, touching or feeling things for a long time)?”**

No. 53 (three months prior to the pandemic) and No. 77 (past two weeks of the survey):

**“How frequently did your child engage in other rituals or routines”**

No. 54 (three months prior to the pandemic) and No. 78 (past two weeks of the survey):

**“Did your child adjust easily to changes in daily routines (e.g., changes in time, location, order, or occurrence of regularly scheduled or typical daily activities such as appointments, mealtimes, or the addition of unexpected events/activities)?”**

No. 55 (three months prior to the pandemic) and No. 79 (past two weeks of the survey):

**“Did your child require family members and others he/she interacts with to maintain specific routines, rituals, habits, including doing things consistently, and requiring warning or change in family behavior (e.g., takes longer to complete tasks, changes schedule to accommodate child)?”**

No. 56 (three months prior to the pandemic) and No. 80 (past two weeks of the survey):

**“Did your child engage in an activity related to a highly restricted, strong interest (e.g., play with the toy/topic, talk about the toy/topic, watch content related to that toy/topic)?”**

The answers for these questions were formulated in a Likert-type format: (1) “not at all,” (2) “rarely,” (3) “occasionally,” (4) “often,” and (5) “regularly.”
